# Women in gig economy work less in the evenings

**DOI:** 10.1038/s41598-022-12558-x

**Published:** 2022-05-19

**Authors:** Sofia Dokuka, Anastasia Kapuza, Mikhail Sverdlov, Timofey Yalov

**Affiliations:** 1 Institute of Education, HSE University, Moscow, Russia; 2 Skyeng Group, Larnaca, Cyprus

**Keywords:** Psychology, Human behaviour

## Abstract

Women have been systematically disadvantaged in the labour market. This could be explained by a complex association of factors, such as the lower speed of women’s professional growth within companies, their under-representation in management positions, and the unequal distribution of caregiving and housework between men and women. The rise of the gig economy—a market system that is based on hiring independent contractors and freelance workers as opposed to creating full-time contracts—has brought researchers and policymakers into a discussion on the effects of online platforms and flexible work arrangements on labour market gender parity. In this study, we examine the case of the largest online English-language school in Eastern Europe, Skyeng. Data on 6,461,404 lessons given by 13,571 teachers demonstrate that women had fewer working hours than men in most age categories, but especially for ages 30–35. The workload deficit for the women could be partly attributed to the fact that they worked less often than the men did in the evenings (7–10 p.m.). We conclude that, despite the flexible work arrangements the gig economy has offered, the women taught fewer classes than the men (i.e., having fewer paid working hours), which in turn led to a gender pay gap. The rapid growth of the gig economy makes it important to monitor gender-gap dynamics as well as discuss potential mechanisms eliminating gender inequality in the labour market.

## Introduction

Women have encountered systematic disadvantage in the labour market. The gender pay gap in the EU (which measures the difference between average gross hourly earnings of male paid employees and of female paid employees as a percentage of average gross hourly earnings of male paid employees) has slightly decreased from 15.8 in 2010 to 13.0 in 2020 although it still shows a persistent pattern of inequality in earnings^1^.

This disparity has been rooted in the complex interaction of multiple factors on individual as well as structural levels. Women have tended to move less rapidly and effectively than men up the career ladder. This gap could be explained both by the over-representation of women in jobs lacking promotion possibilities (so-called “dead-end” jobs)^2,3^, and by their chances of reaching higher hierarchical positions within organizations (the so-called “glass-ceiling effect”)^3^. Women have tended to occupy less privileged industries with low potential for professional growth^4^ such as education, health care, and social work^5^, while remaining under-represented in prestigious fields such as science and technology^6,7^.

Gender disparity in the labour market has gone hand-in-hand with the inequitable distribution of unpaid activities such as caregiving and housework^8^. Although men in many Western societies have increased their engagement in caregiving and housework in recent decades^9,10^, women have continued to work more than men pro-bono^11–15^.

Such unequal distribution in work duties has resulted in a documented deficit of paid working hours for women. On average in the EU, men worked 15% more hours per week than women in the year 2020, at 39.5 h per week for men and 34.1 h for women^16^. The demand for caregiving and housework has made women less flexible: having often engaged in part-time jobs, they have not tended to seek extra working hours or do paid work between the hours of 5 p.m. and 4 a.m.^17^. Scholars also point to the fact that work–life balance problems for men and women differ^18,19^, since women tend to devote more time and resources to the family, while men tend to concentrate on their careers. ‘Dropping dead from career-driven stress, or shriveling emotionally from never seeing one’s children, is a different issue from exhaustion because of the double shift, or not getting promotion because of career interruptions’^18^.

These factors have exacerbated gender inequality, with various negative consequences for women. Due to the central role of labour market positions in individuals’ income and well-being^20,21^, not only has the gender gap made women economically dependent on their spouses and families, but also limited women’s access to societal rights associated with paid labour in the long term^22–24^. As a response, many developed countries have provided special programs aimed at gender parity, e.g., quota systems^25–27^ and paid parental leave for fathers^28^.

Moreover, the worldwide labour market has been transforming due to the emergence and dissemination of the “gig economy”, which could come to affect the gender gap. The Cambridge English Dictionary^29^ has defined *gig economy* as a simple and straightforward “way of working based on people having temporary jobs or doing separate pieces of work, each paid separately, rather than working for anyone employer”. Scholars have characterized the gig economy as short-term work with minimal contractual engagement^30^.

The global gig economy has influenced industries ranging from taxi driving (Uber) to science and software engineering (Upwork), and more workers than ever have been selling their short-term services to clients rather than holding single full-time jobs^31^. Such working arrangements offer a source of easily accessible supplementary income via the performance of small tasks online at a time and place convenient to the worker. The Online Labour Index aimed to measure the supply and demand of online freelance work (as a part of gig economy) shows that demand for such work has increased significantly^32^. In early 2021, almost 90% more projects were demanded via online freelance platforms than in mid-2016, which equals an annual growth rate of 10%.

The effects of the gig economy on the gender gap in the labour market are still unclear. Some scholars have suggested that gender differences in career development, as well as the gender wage gap, could decrease due to flexibility in working arrangements^33,34^. However, previous empirical evidence on various domains of the gig economy did not lead us to such optimistic conclusions. Analyzing Uber drivers’ salaries, Cook et al.^35^ demonstrated that women earned 7% less than men. In this study, the authors did not find any gender differences in a taste for specific hours, work intensity, or customer discrimination.

In line with those results, Litman et al.^36^ observed that on Amazon’s online task platform Mechanical Turk, women earned 10.5% less than men per hour of work, largely because women tended to choose tasks that paid less. The wage gap held after they^36^ controlled for age, race, professional experience, and family status. In their study of the UpWork platform, Foong et al.^37^ demonstrated that female workers’ median hourly bill rates were 74% of those of male workers. This gap also could not be entirely explained by online and offline work experience, education level, or job category^37^. But in this study scholars noted that in some job categories, the association between gender and earnings was more complex: women earned more than men overall by working more hours, outpacing the effect of lower hourly bill rates. Adams-Prassl et al.^38^ demonstrated based on Amazon MTurk digital trace and survey data that women on MTurk earn 20% less per hour than men. This gap was the result of differences in the scheduling of work: women were less likely to work on continuous batches of tasks and were more likely to take longer breaks between submitting one task and starting the next. Such work patterns resulted in slower task completion times and subsequently lower earnings. The authors also demonstrated that there was no wage gap for individuals without children whereas the difference in hourly earnings was highest for women with young children. Additionally, according to the survey data, women were more likely to report that domestic responsibilities affect their ability to complete tasks successfully and without interruption.

The data from Spanish freelance platform^39^ demonstrated that women were significantly less likely to be hired for male-type jobs (e.g., software development) but more likely to be hired for female-type jobs (e.g., writing and translation) than equally qualified male candidates. The author of the study suggests this sorting might be related to gender stereotyping, since employers typically contract freelancers for short-term, relatively low-value jobs based on limited information about job applicants. Subsequently, these conditions might trigger the use of cognitive shortcuts about intrinsic gender characteristics linked to different skills and occupations. The author argues that this sorting mechanism is self-reinforcing and might exacerbate gender imbalance.

Thus, the gig economy could prove to be a crucial factor in the future of labour, having provided unprecedented flexibility for workers. Researchers have suggested an increase in interest in such working arrangements among various groups in society^40^. At the same time, empirical studies have tended to demonstrate that women have been earning less than men in the gig economy, a gap that could be attributed to multiple factors. Gender pay gaps have stemmed from complex dynamics^37^, making additional analyses essential to better understand gender disparities in the gig economy.

### Study objectives and research questions

In this article, we study differences in workload between men and women in the largest online English-language school in Eastern Europe, Skyeng. Rather than being fully employed by Skyeng, the language teachers designate their availability, as an example of the gig economy’s flexible working arrangements. Here we consider the gap between teachers’ workload. The workload was difficult to study because of the limited availability of accurate data on employees' exact working hours. Self-reported information about time distribution is usually limited in temporal granularity, might be affected by social desirability bias, and vulnerable to memory error^41–43^. In contrast, digital trace data offer detailed real-time observation of individual actions^43^, as well as enable retrospective data collection. In this paper, we use information about exact paid working hours of online school teachers. Besides the workload, which plays a primary role in their earnings, teachers’ financial compensation in this online school has been a complex function of multiple adjacent factors, such as platform experience and student satisfaction. Since we do not have information on the exact size of teachers’ financial compensation, we focus exclusively on their workload in this paper.

Our analysis addresses a set of three research questions.

Based on previous findings on women’s lower workload in traditional jobs^4,16^, we hypothesize that this tendency might also hold in the gig economy. Conversely, the flexibility online platforms have come to provide through gig economy jobs might diminish the motherhood penalty^33^. Hence, we arrive at our first research question:

RQ1: Are there statistically significant disparities in workload between the male and female gig economy teachers?

Since workload has been directly associated with pay in this type of work, the answer to RQ1 could help explain the gender pay gap^30^. Less is known about changes in the gender gap over individuals’ life cycles.

Based on data from the US Census and the American Community Survey, Goldin^33^ demonstrated that while men and women began their employment with relatively similar earnings, the gender pay gap soon widened in men’s favor, then narrowed as individuals reached their forties. In other words, the disparity in earnings between male and female workers significantly increased during their first few decades of working life. This gap could be related to a lack of human capital accumulation during the childbirth period^44^. In traditional jobs, white-collar women reported higher levels of stress than men, and this level increased with the number of children. Moreover, the peak of this stress was reached in the period of 35–39 years^45^. Gupta and Smith^44^ suggested that “flexible work-time scheduling, part-time work, home-based work or work-sharing during leaves may be solutions to the problems of depreciation of human capital and the loss of human capital accumulation associated with leave periods”.

In the present study, we gauge the gender gap among gig economy workers in different age groups, which leads to our second research question:

RQ2: Are there statistically significant disparities in workload among the men and women in different age groups?

Finally, workload disparities between men and women could be related to time arrangements during the workday. Unlike traditional work, the gig economy theoretically allows individuals to organize their work hours conveniently around their routine. Women have been less likely than men to work evenings due to caregiving and housework responsibilities^4,15^, and the gig economy gives them the opportunity to work at more suitable times. Our third research question is thus as follows:

RQ3: Are there statistically significant disparities in the distribution of work hours between men and women?

In this article, we consider the data on more than 6 millions English lessons given to adult students over the 78-weeks period from January 1, 2019, to July 1, 2020. We analysed only completed lessons (87.2% of all planned lessons). The data includes information regarding teachers’ self-reported gender and birth date. The Skyeng information system also gathers information on teachers’ time zones.

### Data

Skyeng is the largest online English-language school in Eastern Europe (https://skyeng.eu/). It offers one-on-one, 50-min online lessons. Anyone can sign up as a client at Skyeng.

The Skyeng recommendation system matches students and teachers based on their time preferences (adjusted to time zones) and language level. Students and teachers might be in different time zones. The system offers students a small pool of teachers from which to select. Either the teacher or student may cancel individual lessons or stop lessons altogether.

Admitted teachers have to open at least 12 hourly slots for the lessons at registration. However, they are allowed to adjust their workload and working hours accordingly afterward.

This article covers automatically collected log data on English lessons given to adult students from January 1, 2019, to July 1, 2020. Our data set consists of 6,461,404 lessons given by 13,571 teachers to 216,285 students. We considered completed lessons only (87.2% of all planned lessons). We included information regarding teachers’ self-reported gender and birth date. The Skyeng system also gathers information on teachers’ time zones.

Skyeng hires both native and non-native English speakers for teaching positions. Teachers were mostly based in Russia (49.23%) and former USSR countries (15.13% in Ukraine, 10.30% in Kazakhstan, 6.12% in Armenia, etc.). The rest of the teachers (~ 8%) were based in Europe, the US, and other countries. Their mean age varied from 28 to 30 during the observation period.

## Results

**Figure 1:**
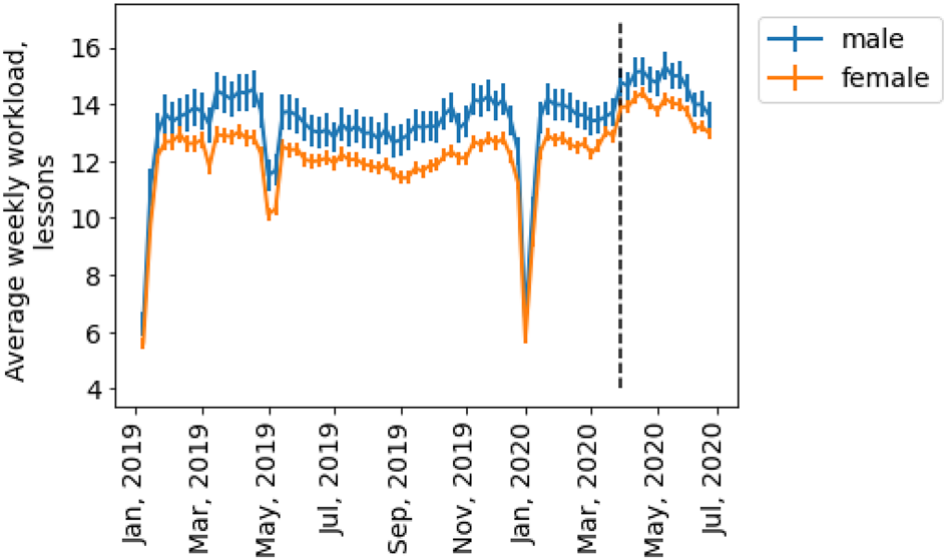
Average weekly teaching workload over 78 weeks, from January 1, 2019, to July 1, 2020. Vertical bars correspond to 95% confidence intervals. The black dashed line indicates the start of the national lockdown. The male teachers had a higher workload than the female teachers at Skyeng. Workload decreased in January and May (New Year and Christmas and national holidays, respectively).

The dynamics of the average weekly workload for the male and female teachers in our data set are presented in Fig. 1. We found four points of decline in such dynamics, namely weeks 1, 18, 19, and 53, which corresponded to Russian national holidays in January and May. While we observed a rapid decrease in the number of lessons given in May 2019, the same did not happen in 2020, which could be due to the impact of the COVID-19 pandemic on teaching and learning strategies and on the online work structure. Despite the national holidays, students did not pause their studies at the time of the nationwide COVID-19 lockdown that started on 30 March 2020 in Russia.

To address disparities in the workload of the men and women, we excluded the four weeks corresponding to the national holidays in May 2019 and January 2019 and 2020. The Skyeng teachers were found to have given an average of 12.83 lessons per week (95% CI [12.80; 12.85]) during the observation period. The mean workload for the men was higher than for the women by almost 8.40% (x_men_ = 13.76, x_women_ = 12.61, p-value < 10^–4^, Welch’s t-test). Thus, we concluded that the female teachers, on average, worked fewer billable hours than the male teachers (RQ1).

**Figure 2:**
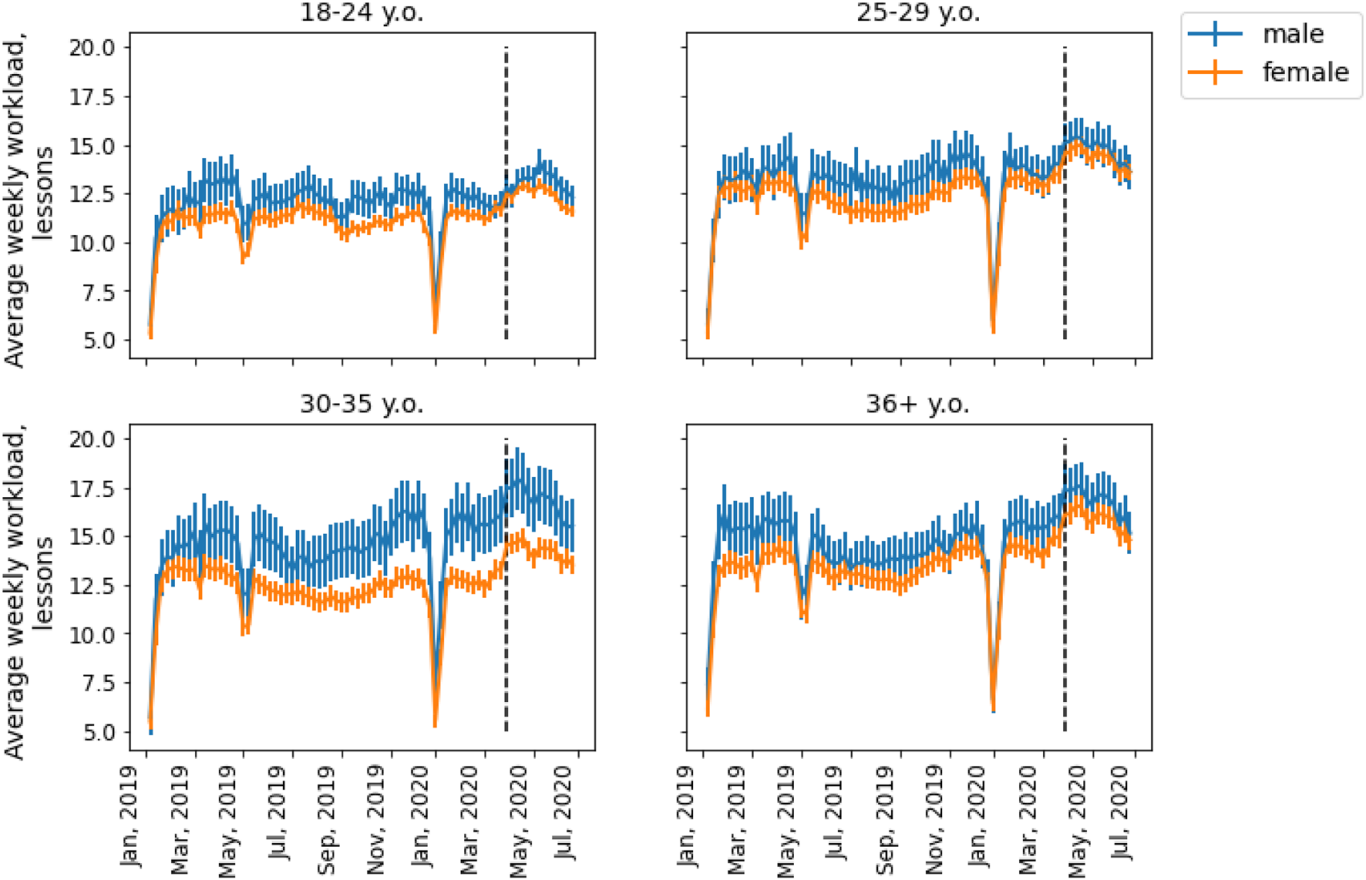
Average weekly teaching workload over 74 weeks, from January 1, 2019, to July 1, 2020, for four age categories. Vertical bars correspond to 95% confidence intervals. The black dashed line corresponds to the start of the national lockdown in Russia. Skyeng’s male teachers had a higher workload than its female teachers notably in the 30–35 age group.

**Figure 3:**
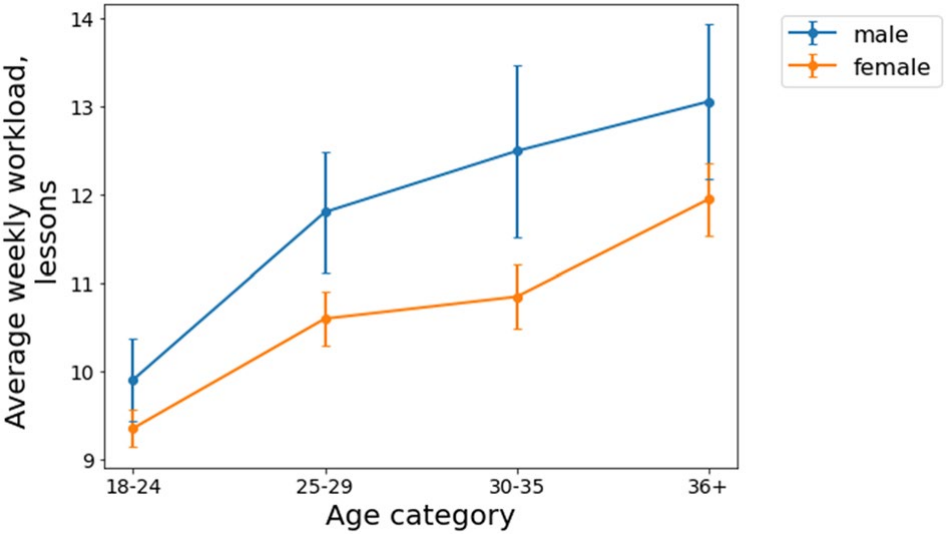
Average weekly teaching workload for four age categories in 2019. Vertical bars correspond to 95% confidence intervals. Skyeng’s male teachers had a statistically significant higher workload than female teachers in each of the age categories.

To gauge the effects of age on workload gender disparity, we examined teachers’ workload by different age groups. The sample was divided into four age categories: 18–24, 25–29, 30–35, and older than 35. The dynamics of the average weekly workload for the male and female teachers in each of these groups can be seen in Fig. 2. The average weekly workload of teachers from distinct age categories with respect to gender can be seen in Fig. 3, we considered only 2019 for the analysis due to workload shift associated with COVID-19 pandemic. The national holiday weeks were excluded from our data set.

Our results indicate that men’s workload was statistically significantly (*p* < 0.05) higher than that of women for 62.16% of the weeks in the observation period for those aged 18–24; 28.38% for those 25–29; 90.54% for those 30–35; and 29.73% for those older than 35. Pairwise comparisons demonstrate that in each of the four groups women tend to teach less classes that men (the results of pairwise comparisons for different age groups: x_men 18–24_ = 9.35, x_women 18–24_ = 9.90, t = 2.12, *p* = 0.03; x_men 25–29_ = 11.80, x_women 25–29_ = 10.60, t = 3.15, p = 0.002; x_men 30–35_ = 12.49, x_women 30–35_ = 10.85, t = 3.13, p = 0.002; x_men 36+_  = 13.05, x_women 36+_  = 11.95, t = 2.26, *p* = 0.02). Although the male teachers tended to teach more classes than the women overall, it can be said that the workload gap was closely associated with their age (RQ2). For the younger group (18–24) the difference in workload is half an hour, while for the 25–29 group the workload difference is 1.2 h, and for the group of 30–35 the gender difference is at maximum value of 1.6 h. For the oldest group of teachers the workload difference is 1.1 h. Our findings corroborate with previous results on gender gap evolution^33^ that demonstrate the gender difference is quite modest at the onset of one’s career, then it shows a tendency toward widening in men’s favor, and later narrows as individuals reach their forties. Still, we should note that Goldin’s results are based on salaries, while in this paper we focus on employees' workload.

**Figure 4:**
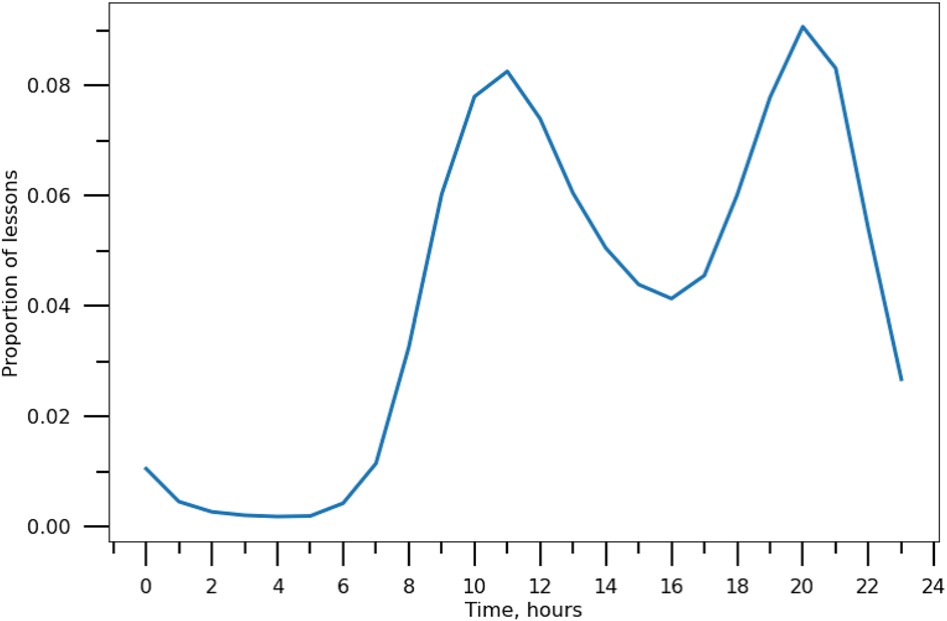
Hourly changes in the proportion of lessons. Skyeng lesson intensity displayed peaks in the morning (10:00 to 13:00) and evening (19:00 to 22:00). The minimal activity level corresponds to the night period (01:00 to 06:00).

Figure 4 demonstrates the presence of diurnal patterns on Skyeng (participants’ time zones were considered). Teachers’ time zones were accounted for in the analysis. Lesson intensity had morning (from 10:00 to 13:00) and evening (from 19:00 to 22:00) peaks. More than a quarter of lessons (25.10%) took place during the evening peak, with a slightly smaller fraction of lessons (22.00%) in the morning peak. By contrast, only 1.72% of lessons were completed during the night hours (from 01:00 to 06:00) corresponding to the period of least activity. The distribution follows the expected pattern of the rhythm, similarly to other online educational resources^41,46^. Traditional employees generally follow different working patterns and prefer to schedule their working hours from 9:00 to 17:00 or from 8:00 to 16:00^47^. Having said that, it is worth mentioning that according to RescueTime study^47^, while a large number of people ended their day between 17:00 and 18:00, nearly 40% continued using their devices after 22:00. Naturally, this does not prove that these individuals worked during these hours, however, we might suggest that at some point their activity might have been related to their work.

**Figure 5:**
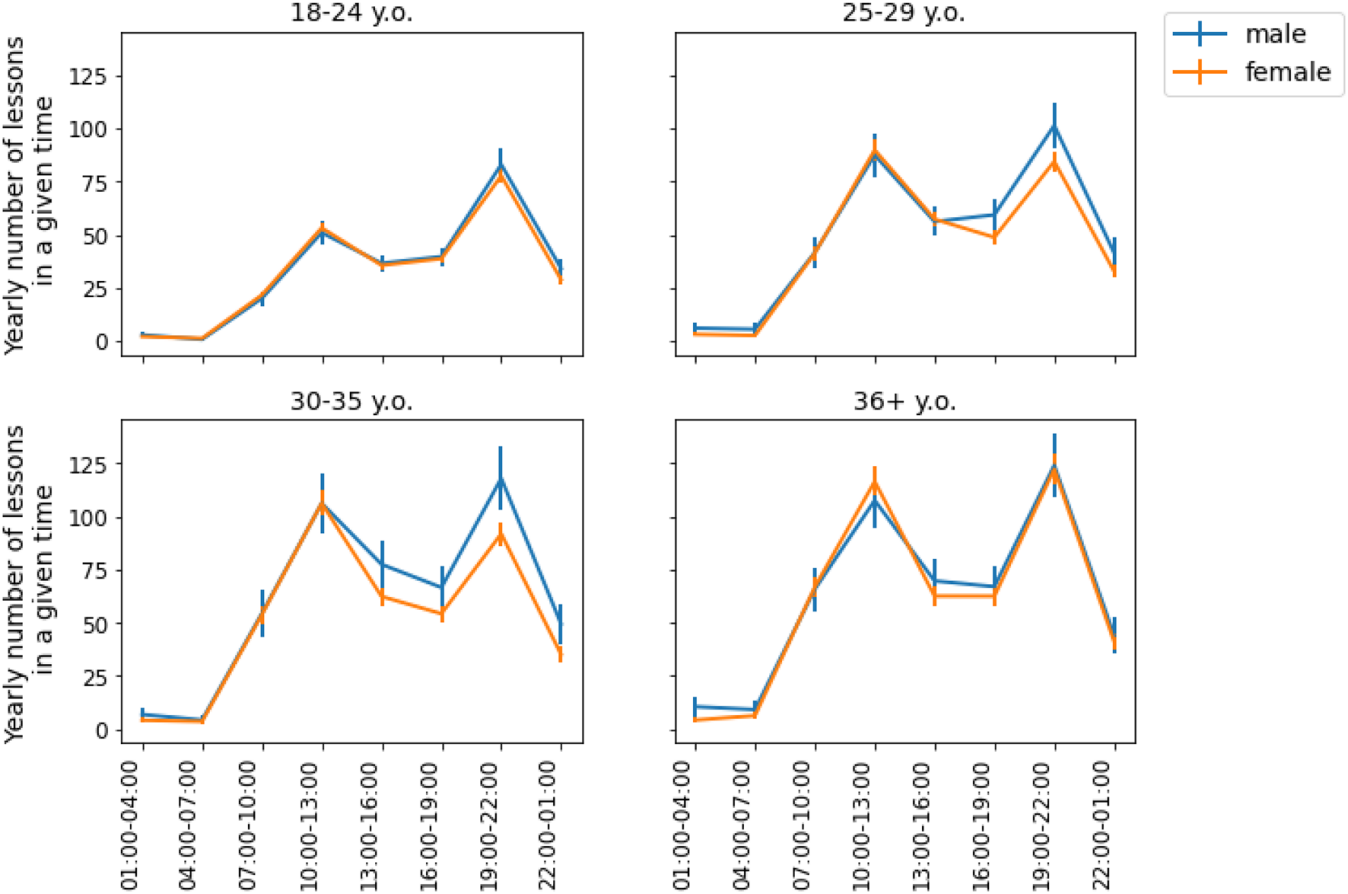
Average yearly teaching workload in 2019 for four different age categories. Vertical bars correspond to 95% confidence intervals. Skyeng's male teachers had a higher workload than its female teachers notably in two age groups: 25–29 and 30–35.

To account for possible shifts due to the COVID-19 lockdown, we analysed data from 2019 and 2020 separately. The average workload for teachers at various times in 2019 is shown in Fig. 5. We found that in two of the age groups (25–29 and 30–35), men tended to give statistically significantly more lessons in the evenings (19:00–22:00) than women (mean number of lessons given: x_men 25–29_ = 101.35, x_women 25–29_ = 84.41, t = 2.86, *p* < 0.01 and x_men 30–35_ = 117.94, x_women 30–35_ = 91.87, t = 3.18, *p* < 0.01, correspondingly).

**Figure 6:**
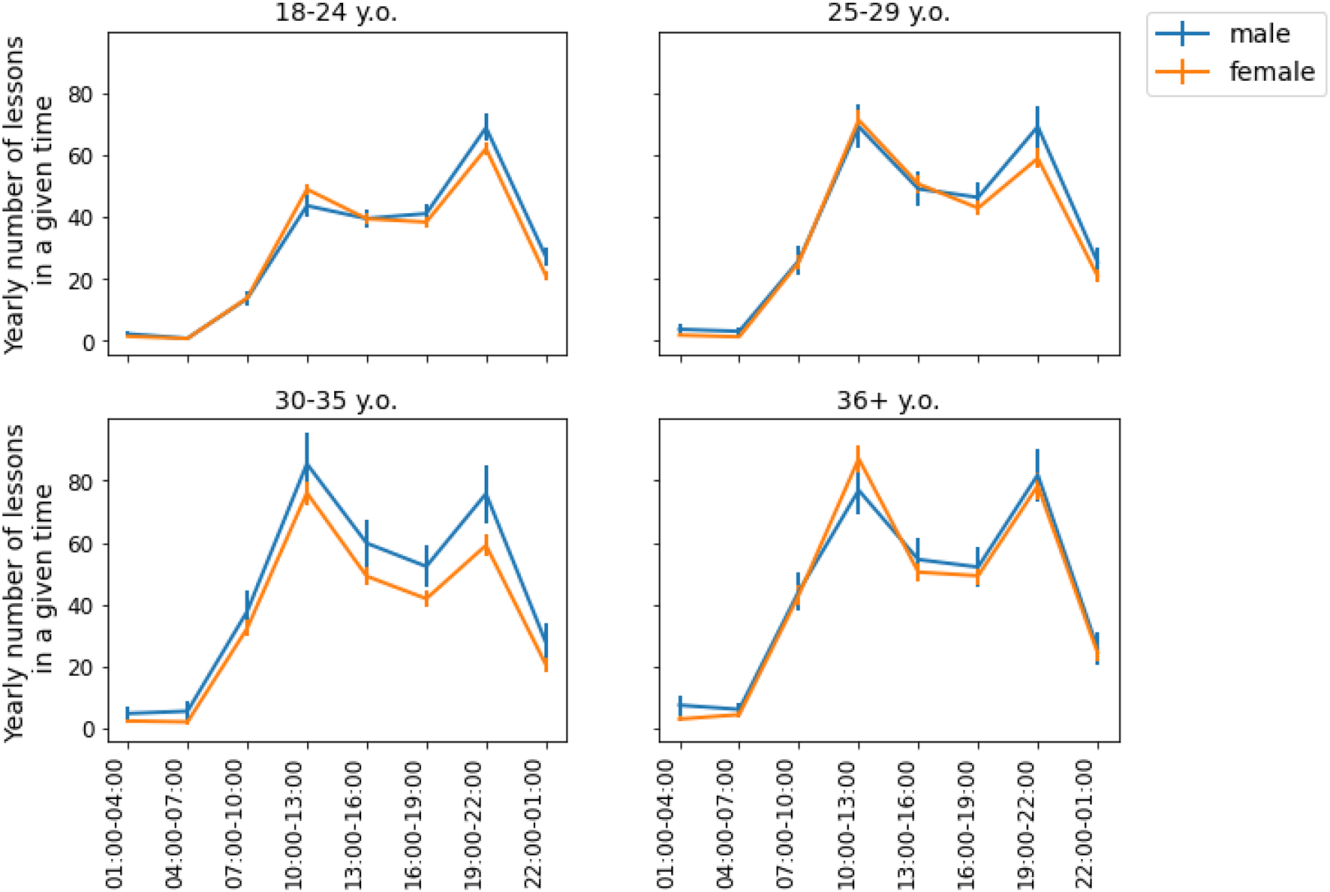
Average yearly teaching workload in 2020 for four different age categories. Vertical bars correspond to 95% confidence intervals. Skyeng’s male teachers had a higher workload than female teachers notably in three age groups: 18–24, 25–29, and 30–35.

This trend remained in 2020 (Fig. 6). While Skyeng teachers’ overall workload increased during the COVID-19 pandemic, the women from the same age group 30–35 gave statistically significantly fewer lessons in the evenings than the men for the whole year (mean number of lessons given: x_men 30–35_ = 75.77, x_women 30–35_ = 59.08, t = 3.30, *p* < 0.01). Moreover, this disparity appeared for other two groups: 18–24 (mean number of lessons given: x_men 18–24_ = 68.79, x_women 18–24_ = 62.03, t = 2.74, *p* < 0.01) and 25–29 (mean number of lessons given: x_men 25–29_ = 69.14, x_women 25–29_ = 58.86, t = 2.75, *p* < 0.01). To summarize, the gender differences in workload that were related to age could be partially attributed to the distribution of work hours (RQ3). We might suggest that these differences may emerge due to the limited availability of women in the evenings, despite evening hours being the most in-demand for students.

## Discussion

Gender inequality has long been an important economic and societal challenge. Quota systems and parental leave for fathers have been implemented in many countries toward achieving gender equality^25,28^. Continuing labour market changes tied to online platform development (the gig economy) have created unique opportunities for flexible working arrangements and home-based work.

This article examined gender inequality in a gig economy case study, in search of potential explanations for the gender workload gap. Using the data on teachers’ workload for the online platform Skyeng, we considered the gender differences in workload and tried to explain it through age and working hours distribution. Although women held most of the teaching positions in the Skyeng online school, the women had fewer working hours than the men. These differences were significant under the age of 35 and smoothed out for older groups. A similar age-related trend has been previously shown in other studies, but it demonstrated the difference in earnings based on data from US Census^44,45^. In this research, we suggest that the observed workload gap could be partially attributed to differences in a workday organization: i.e. women work less during evenings.

Although we did not collect data on teachers’ marital or family status, considering that mothers’ mean age of childbirth in Russia in 2019 was 29^48^, we may be able to associate the lack of workload with these women’s caregiving duties. In line with previous study results^17^, we found that the women, on average, worked less than the men in the evening. We could suppose that these hours were often devoted to childcare and time with family.

It is worth noting that our research was limited to data on activity logs and the personal characteristics of the teachers in our sample. Although this allowed us to track actual teachers' working patterns, we could not detect the causal mechanisms behind the formation of differences. Further survey-based studies, interviews, and focus groups are necessary to shed light on male and female working experiences in the broader gig economy and identify barriers to equal opportunities. Another issue is that we could not control the additional employment among teachers. Additionally, we should take into account that online teaching is not a gender-blind system compared to other tasks and projects within the gig economy. In this research we did not check whether students had any gender preferences, although further research should address this question.

In conclusion, despite the home-office and flexibilization opportunities the gig economy has created, gender disparity has remained significant in this and other labour sectors. Due to various structural and individual-level factors, men have remained dominant in the labour sphere. Additionally, many jobs in the gig economy do not provide opportunities for professional development and career growth and could be considered “dead-end” jobs.

Our findings concur with previous empirical results on the prevalence of a gender gap in the gig economy. The majority of studies on gender imbalance in the labour market were completed based on data from WEIRD (Western, educated, industrialized, rich, and democratic) societies^30,49,50^, which makes it challenging to understand labour market disparity in other societies. In this paper, we demonstrate that the workload gender gap is observed also in the CSI countries (former Soviet Union countries), so gender disparity holds in different societies.

These findings have demonstrated that policymakers, experts, researchers, and the owners of gig economy platforms should develop additional measures to promote gender equality in this fast-growing labor sector. Besides, the implementation of such policies should account for regional specificity.

Our results also highlight that women in the age group 30–35 are the most vulnerable group due to limited working capacity and potentially largest caring duties. Even flexible and distant working arrangements do not prevent the gender gap, especially for this group. Interestingly, recent studies even found that home-based work would be more financially beneficial to men^51^. Additionally, recent research documents this category also lacks in well-being^43,45^ which might be associated with the perceived unequal distribution of household duties, and work-family conflict^52^. Thus, we suggest paying additional attention to the identification of such vulnerable groups, detailed investigation of the multidimensional nature of the gender gap, and moreover, difficulties of women’s development. Furthermore, we recommend to develop targeted programs aimed at the support of such risk categories.

Additionally, we would like to accentuate the effects of COVID-19 pandemic on gender inequality in the gig economy. The pandemic was associated with both the growth of the gig economy (i.e., an increase in the number of average daily tasks/jobs posted on digital platforms^53^) and challenges for women’s advancement and professional development^54^. We argue that these shifts in the work landscape in the gig economy require attention and analysis.

Social change facilitated by the spread of gig economy has an impact on a variety of human life spheres besides the labour market. One of them is the environmental factor. The majority of gig economy workers complete their tasks in a remote regime on their own devices (e.g. in the case of Skyeng teachers they give lessons from their computers) and they have no need to commute. Common sense says that such a working organization has a lower environmental impact than in-person office working style. Besides, in contrast to traditional employment, the gig economy does not require employees’ in-person presence; consequently, there is no need for them to commute. Such remote working might have a positive impact on global emission. According to D’Almeida et al.^55^ transport accounts for 40% of global emissions, and a large share of transport emissions emanates from commuting. Agent-based modeling demonstrated^56^ that by intensifying remote work to 2 or 3 days a week, nitrogen dioxide concentrations are reduced, on average, over 4% and 8%, respectively. Notably, remote work might be a key tool in alleviating traffic congestion during peak time in addition to air quality improvement. Although distant employment might be considered as an instrument toward sustainability, some scholars suggest that such working organization might have two-fold environmental effect. Shreedar et al.^57^ considered a set of environmentally relevant individual behaviour effects of working from home on energy consumption, travel, technology use and waste behaviour. They outlined the duality of the remote regime on sustainability. For instance, remote workers might be active in their non-working travel which is associated with larger emissions. Alternatively remote workers might be more active digital technology users which is also associated with larger emission. Additionally, gig economy employers cannot control the quality of the laptops or PCs that individuals use for their work. This discussion shows that growth of the gig economy and new forms of employment and working impact a wide range of life spheres and it is important to empirically analyze the evolution of the labour market from different perspectives.

## Methods

Skyeng is the largest online English-language school in Eastern Europe (https://skyeng.eu/). All lessons are conducted via the Vimbox platform. The lessons are standardized and the teachers, as well as the students, are provided with all the related materials.

Skyeng hires both native and non-native English speakers for teaching positions. In the case of non-native speakers, the school prefers to hire teachers who have TEFL, CELTA, or DELTA certificates. Job-seekers must complete an audio interview and give a test lesson. Successful candidates must also complete a short probatory period. On admission teachers have to open at least 12 hourly slots for the lessons in their schedule. However, they are allowed to adjust their workload and working hours accordingly afterward.

The payment for the lesson is fixed by the platform. A student buys a package of lessons and pays only for their number. The teacher's remuneration is made for each lesson and directly depends on the number of lessons in a given period. When the lesson is finished, the teacher’s payment is transferred to a virtual account. The teachers can withdraw the money from the virtual account to their bank account biweekly.

Any person in the world is able to register on the Skyeng platform as a student. Upon registration on the platform, students have to pass an admission interview with the manager in order to assess the level of English language proficiency, set the study goals, identify personal interests and find out the time preferences. Afterward, the Skyeng automated recommendation system provides a student with a set of available teachers to select from. For each teacher, the system shows a teacher's photo, personal interests, location and a short bio in text and audio formats.

Both students and teachers are able to cancel and/or postpone separate lessons and/or terminate their partnership at any time.

This article covers automatically collected log data on English lessons given to adult students from January 1, 2019, to July 1, 2020. Our data set consists of 6,461,404 lessons given by 13,571 teachers to 216,285 students. We considered completed lessons only (87.2% of all planned lessons). We include information regarding teachers’ self-reported gender and birth date. The Skyeng system also gathers information on teachers’ time zones.

**Figure 7:**
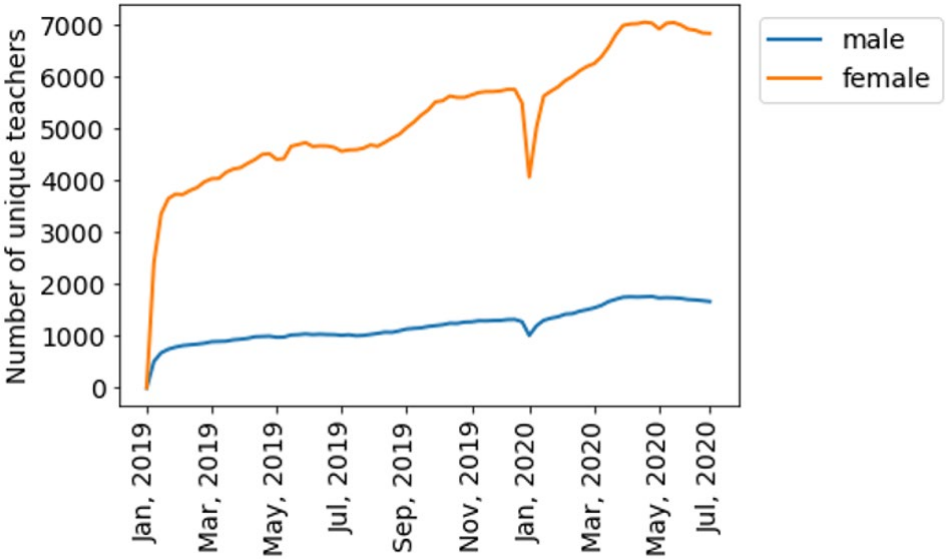
The dynamics of the number of unique teachers (men and women) during the observation period.

**Figure 8:**
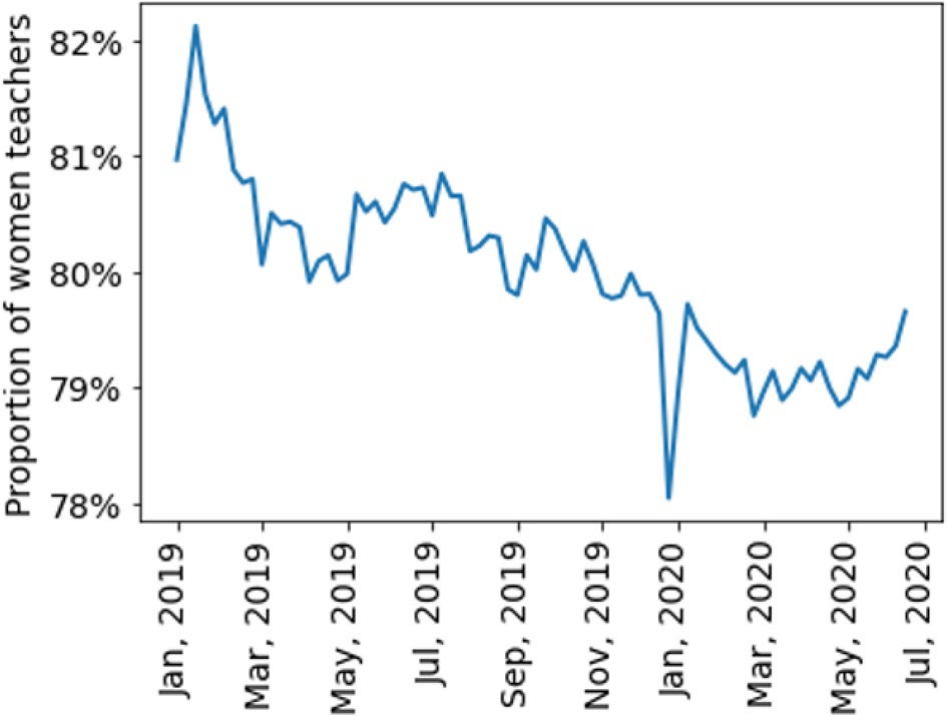
The proportion of women in the sample during the observation period.

**Figure 9:**
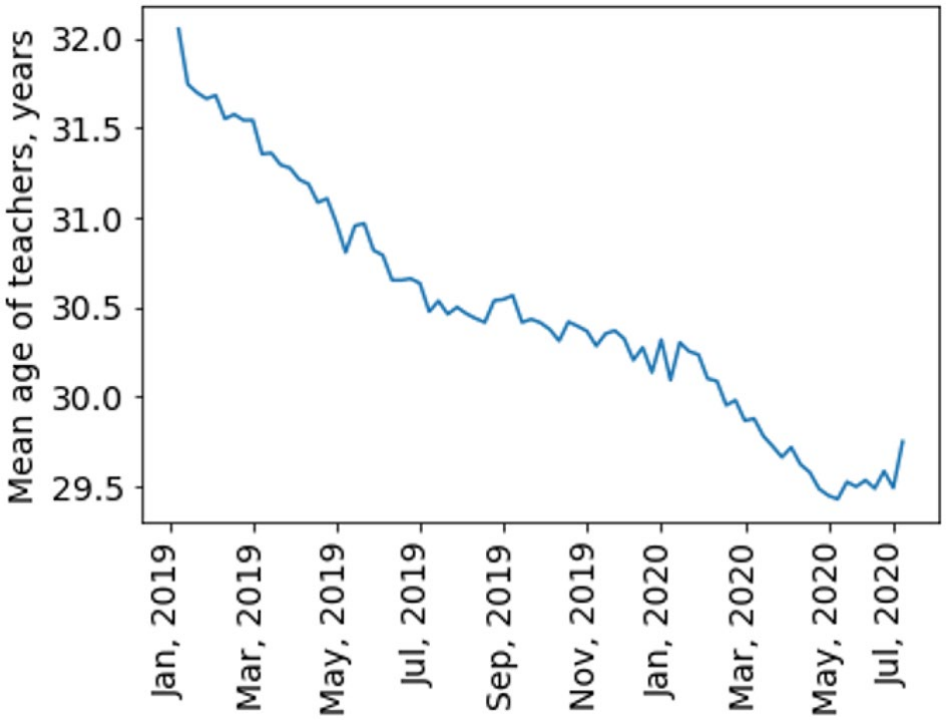
The dynamics of mean age of Skyeng teachers.

Figure 7 shows the dynamics of the absolute number of unique teachers in the data set. Most of the teachers were female (80%) and this proportion slightly changed over time (Fig. 8). Information of age with respect to teachers’ gender is presented in Table 1. Their mean age varied from 29 to 32 during the observation period (Fig. 9). More than half of the teachers (58.34%) were located in GMT + 3 time zone which corresponds to Moscow, Saint-Petersburg and central Russia. Ten percent (10.29%) of the teachers were in GMT + 4 time zone which corresponds to Samara. Almost ten percent (9.84%) of the teachers reported living in GMT + 6 time zone which corresponds to Novosibirsk and Altay regions. The rest of the sample reported living in other time zones corresponding to Russia, Europe and America.

**Table 1:** The number of teachers in different age categories with respect to their gender (values were computed at 2019, January).

	18–24 y.o	25–29 y.o	30–35 y.o	36 + y.o
Male	1174	583	358	413
Female	4671	2335	1810	1679

Computations were performed in pandas package^58^ (Python 3.8^59^). We used matplotlib^60^ for visualizations. All tests for equal means in this article were completed by Welch’s t-test to account for unequal size and variance between paired samples.

### Ethical declaration

The study was approved by the HSE Committee on Interuniversity Surveys and Ethical Assessment of Empirical Research. An individual informed consent for the study was not required, since at the beginning of the platform use, all teachers and students signed an offering statement that Skyeng may share collected data with third parties for scientific purposes. We confirm that all methods were performed in accordance with the relevant guidelines and regulations.
